# Revaccination of Cattle with Bacille Calmette-Guérin Two Years after First Vaccination when Immunity Has Waned, Boosted Protection against Challenge with *Mycobacterium bovis*

**DOI:** 10.1371/journal.pone.0106519

**Published:** 2014-09-02

**Authors:** Natalie A. Parlane, Dairu Shu, Supatsak Subharat, D. Neil Wedlock, Bernd H. A. Rehm, Geoffrey W. de Lisle, Bryce M. Buddle

**Affiliations:** 1 AgResearch, Hopkirk Research Institute, Palmerston North, New Zealand; 2 Institute of Fundamental Sciences and MacDiarmid Institute for Advanced Materials and Nanotechnology, Massey University, Palmerston North, New Zealand; 3 AgResearch, National Centre for Biosecurity and Infectious Disease - Wallaceville, Upper Hutt, New Zealand; Public Health England, United Kingdom

## Abstract

In both humans and animals, controversy exists concerning the duration of protection induced by BCG vaccine against tuberculosis (TB) and whether revaccination enhances protection. A long-term study was undertaken to determine whether BCG-vaccinated calves would be protected against challenge with *Mycobacterium bovis* 2½ years after vaccination and to determine the effect of revaccination after 2 years. Seventy–nine calves were divided into five groups (n = 15–17 calves/group) with four of the groups vaccinated subcutaneously with 10^5^ CFU of BCG Danish at 2–4 weeks of age and the fifth group serving as non-vaccinated controls. Three of the four BCG-vaccinated groups were revaccinated 2 years after the initial vaccination. One BCG-vaccinated group was revaccinated with BCG. A second group was vaccinated subcutaneously with a TB protein vaccine consisting of biopolyester particles (Biobeads) displaying two mycobacterial proteins, ESAT-6 and Antigen 85A, mixed with an adjuvant. A third group was vaccinated with TB proteins from *M. bovis* culture filtrate, mixed with an adjuvant. Twenty-three weeks after the BCG revaccination, all animals were challenged endotracheally with virulent *M. bovis* and a further 13 weeks later, animals were killed and necropsied to determine protection against TB. The BCG-vaccinated animals produced positive tuberculin caudal fold intradermal (15 of 62 animals) and IFN-γ TB test responses (six of 62 animals) at 6 months after vaccination, but not at subsequent time-points compared to the non-vaccinated animals. Calves receiving a single vaccination with BCG vaccine 2½ years prior to challenge were not protected against TB, while those revaccinated with BCG 2 years after the initial vaccination displayed significant reductions in lung and pulmonary lymph node lesion scores compared to the non-vaccinated animals. In contrast, no reduction in lesion scores was observed in the animals revaccinated with the TB protein vaccines with their immune responses biased towards induction of antibody.

## Introduction

Bovine tuberculosis (TB) caused by *Mycobacterium bovis* constitutes a major animal health problem in many countries and improved control strategies are urgently required. Although, the implementation of “test and slaughter” control programmes have resulted in bovine TB being eradicated from a number of countries [1], these measures have been less effective in countries which have wildlife reservoirs of *M. bovis* infection or where these programmes are economically or socially not acceptable. Currently, there is renewed interest in the use of TB vaccines for cattle from the realisation of the financial impact of bovine TB on animal health and trade and also due to the difficulty of control of the disease. There are no TB vaccines licenced for use in cattle, although BCG vaccine has been shown to induce significant levels of protection against experimental challenge with *M. bovis* in short term trials and in recent field trials (reviewed in [2]). The major caveats which have restricted BCG being used in cattle until now have been that protection may not be complete and vaccination sensitises animals to respond in routine TB diagnostic tests.

The diagnostic issue can be overcome by using new tests which can differentiate infected from vaccinated animals (DIVA tests) using antigens which are not expressed by BCG [3], [4]. However, widespread use of these tests may be constrained by economic or practical considerations. Encouragingly, tuberculin intradermal test responses have been shown to rapidly decline between 6–9 months after BCG vaccination when measured by the single intradermal comparative cervical tuberculin test (SICCT) [5]. Further studies are now required to determine whether a similar decline in tuberculin intradermal test sensitivity is observed in cattle which are tuberculin-tested using the intradermal caudal fold test (CFT). This test is undertaken in countries in the Southern hemisphere and North America as it is more cost-effective and practical for the yarding conditions which exist in these regions. For this test, bovine tuberculin is inoculated intradermally into the caudal fold of the tail, in contrast to the SICCT where responses to bovine and avian tuberculin are compared following intradermal inoculations in the neck.

To improve the efficacy of BCG vaccination of cattle, insights can be gained from studying the use of BCG vaccine in humans. Conversely, cattle can also be a useful model for improving BCG use in humans [6], [7]. It is recognised that BCG vaccination provides protection against childhood tuberculous meningitis and miliary TB, although efficacy against pulmonary TB in children and adults is highly variable, ranging from 0 to 80% [8]. The higher efficacy is seen in individuals with no detectable prior exposure to environmental mycobacteria (negative tuberculin intradermal test response) [9]. In humans, there is continuing debate concerning the duration of immunity induced by BCG vaccination and whether revaccination with BCG is effective. Where BCG is protective, the duration of protection is variable, averaging about 10 years [10], although protection lasting 40–50 years has been observed in American Indians and Alaskan natives [11], [12]. Revaccination of older children and adults has not been recommended by WHO as it is thought to be neither sufficiently protective nor cost-effective [13], [14], although a recent analysis by Dye [9] has questioned this view.

A recent study in cattle investigated the duration of protection induced by BCG vaccination and showed that a significant level of protection was induced when cattle were experimentally challenged with *M. bovis* 12 months after vaccination, but not after 24 months [15]. It is possible that a limited protective effect was present up to 24 months as lesion scores were 20% lower than those in the non-vaccinates, but these did not reach significance due to relatively small group sizes. Larger group sizes are required to answer this question as well as to determine whether protection can be enhanced by revaccination with BCG.

A strategy being pursued in the development of improved human vaccines has been to prime individuals with BCG and later boost immunity with either TB protein or virus-vectored vaccines expressing mycobacterial antigens [16]. Vaccination studies in cattle have shown that co-adminstration of BCG and a TB protein vaccine consisting of *M. bovis* culture filtrate protein mixed with an adjuvant have induced better protection than BCG alone [17], [18]. Revaccinating BCG-vaccinated cattle with a TB protein vaccine could be a means to enhance longevity of protection.

The aims of the current study were to establish how long BCG vaccination interfered with the interpretation of the tuberculin intradermal CFT and the whole blood interferon-γ (IFN-γ) test, whether BCG-vaccinated calves were protected against a TB challenge after 2½ years and if protection was enhanced by revaccination with BCG or with TB protein vaccines. The two TB protein vaccines comprised a *M. bovis* CFP vaccine [17], [18] and a TB protein vaccine consisting of biopolyester particles (Biobeads) displaying two mycobacterial proteins, ESAT-6 and Antigen 85A (Ag85A) which has been shown to induce protection in a mouse TB model [19].

## Materials and Methods

### Ethics statement

All animal procedures were approved by AgResearch Grasslands Animal Ethics Committee; permit number 12413.

### Animals

Seventy-nine Friesian-cross, Ayrshire and Jersey, female calves, 1-week old were obtained from herds which were accredited as TB-free and from areas of New Zealand where both farmed and feral animals were free of TB. The calves were fed pooled colostrum for the first 4 weeks and then maintained on whole milk for a further 4 weeks. Meal was provided for the calves from 1 to 10 weeks of age, until they were weaned onto a pasture-only diet. The calves were kept on wood shavings for the first 5 weeks and on pasture thereafter. Immediately prior to the cattle being challenged with *M. bovis* at 2½ years of age, they were moved to a biocontainment unit where they grazed on pasture and crops.

### Bacterial strains and vaccines

The lyophilised *M. bovis* BCG Danish 1331 vaccine (Statens Serum Institute, Copenhagen, Denmark) formulated for human use was used to vaccinate the calves. Each vial of vaccine contained 2–8**×**10^6^ CFU of BCG as specified by the manufacturer within the specified shelf life. *M. bovis* strain 83/6235, originally isolated from a tuberculous possum in New Zealand, was used as the challenge strain and had been used in previous vaccination/challenge studies in cattle [17], [20]. Bacteria were grown to mid-log phase in Tween albumin broth (Dubos broth base, Difco Laboratories, Detroit, MI, USA) supplemented with 0.006% (vol/vol) alkalinized oleic acid, 0.5% (wt/vol) albumin fraction V and 0.25% (wt/vol) glucose. Dilutions were made in Tween albumin broth to obtain the dose for inoculation. The number of CFU inoculated was determined retrospectively by plating 10-fold dilutions on Middlebrook 7H11 (Difco) supplemented with 0.5% (wt/vol) albumin, 0.2% (wt/vol) glucose and 1% (wt/vol) sodium pyruvate as previously described [20].

### Preparation of vaccines and vaccination

The BCG vaccine was prepared by reconstituting the vial of vaccine in 1****ml of Sauton medium (Statens Serum Institute) and diluted 1∶4 in phosphate buffer saline (PBS). The 0.5****ml dose of the diluted vaccine contained 2 to 8**×**10^5^ CFU of BCG and represented 1/10 of the BCG in the vaccine vial. The TB biobead protein vaccine (Biobeads) consisted of biopolyester nanoparticles (50–300****nm in diameter) displaying mycobacterial antigens, Ag85A and ESAT-6 as a fusion protein, on their surface and were produced in *Escherichia coli* [19]. The 2****ml dose of vaccine contained 200** µ**g of fusion protein (40% of which was mycobacterial proteins), 250** µ**g of Pam_3_CSK_4_ (EMC Microcollections, Tuebingen, Germany) and mixed in Emulsigen adjuvant (30% final volume; MVP Laboratories, Omaha, NE, USA). The *M. bovis* culture filtrate protein vaccine (CFP) was prepared from a culture of *M. bovis* AN5 as previously described [21]. The 2****ml dose of this vaccine contained 400** µ**g *M. bovis* culture filtrate protein, 250** µ**g Pam_3_CSK_4_ and mixed in dimethyldioctadecyl ammonium bromide (DDA; Sigma Chemicals, St. Louis, MO, USA). DDA was prepared by heating a 10****mg/ml solution at 80°C until micelles formed, cooled to room temperature and added 1∶1 to the rest of the vaccine constituents.

For the initial vaccination with BCG, the calves were divided into two groups by using a stratified random sampling system such that both groups had a similar distribution of breeds. One group contained 62 calves and the other 17 animals. When the calves were between 2–4 weeks of age, the larger group of calves were vaccinated subcutaneously in the mid-cervical region on the left side of the neck with a 0.5****ml dose of the diluted BCG vaccine. The group of 17 calves served as non-vaccinated controls (negative control). Approximately 2 years later (106 weeks after first vaccination), the 62 BCG-vaccinated cattle were divided into four groups, again using a stratified random sampling system for breed distribution, with each group containing 15 to 16 animals. One group of BCG-vaccinated cattle (n = 16) was not revaccinated (BCG once group), while a second group (n = 15) was revaccinated with BCG vaccine in a similar manner as undertaken previously (BCG/BCG group). Cattle from the third group (n = 15) were vaccinated subcutaneously in the neck with a 2****ml dose of the TB Biobead vaccine (BCG/Biobeads group) and cattle from the fourth group (n = 16) received *M. bovis* CFP vaccine administered in a similar volume and manner (BCG/CFP group). The calves in BCG/Biobeads and BCG/CFP groups were reinoculated with the same TB protein vaccine 3 weeks later.

### *M. bovis* challenge and necropsy procedure

The calves were challenged endotracheally with 5**×**10^3^ CFU of virulent *M. bovis* at 2½ years of age (23 weeks after revaccination) as previously described [20]. All cattle were killed 13 weeks after challenge. Procedures for identifying macroscopic tuberculous lesions and processing for histology have been described previously [20]. A lung lesion score was calculated by counting the total number of lesions and applying a score as follows: 0, no lesions; 1, 1–9 lesions; 2, 10–29 lesions; 3, 30–99 lesions; 4, 100–199 lesions; 5, ≥200 lesions. A total lymph node lesion score per animal was calculated by pooling scores for four pulmonary lymph nodes (left and right bronchial and anterior and posterior mediastinal). Scores for individual lymph nodes were: 0, no lesions; 1, 1–19 small lesions (1–4****mm diameter); 2, ≥20 small lesions (1–4****mm diameter) or medium size lesion(s) (5–9****mm diameter); 3, large lesion(s) (≥10****mm diameter). Samples from the four pulmonary lymph nodes were collected from all of the animals for bacterial culture to confirm *M. bovis* infection and for histological examination. Additional samples were collected from any tuberculous-like lesions observed in the lungs. For bacterial culture, tissue samples were homogenized in a Tenbroeck grinder (Wheaton, Millville NJ, USA), decontaminated in 0.75% cetylpyridium chloride for 1 hours, centrifuged at 3500×*g* for 20****minutes and processed for isolation of mycobacteria as described previously [20]. For histological examination, sections were stained with hematoxylin and eosin. Scoring of histopathological lesions in the pulmonary lymph nodes was based on the scale described by Wangoo et al. [22]. Briefly, Stage I granulomas were composed of accumulations of epitheloid macrophages with low numbers of lymphocytes, neutrophils and Langhans multinucleated giant cells and there was an absence of necrosis. Stage II granulomas were similar to Stage I granulomas but also had central infiltrates of neutrophils and lymphocytes and necrosis could be present. Stage III granulomas exhibited complete fibrous encapsulation and significant necrosis and mineralisation could be present. Stage IV granulomas were characterised by multiple coalescing caseo-necrotic granulomas with multicentric necrosis and mineralisation. When no granulomas were observed, the tissue section was scored as Stage 0. The histopathological score was based on the most severe lesion observed in each pulmonary lymph node section with scores ranging from 0 to 4, corresponding to Stages 0 to IV. A total histopathological score was compiled by pooling scores for each of the four pulmonary lymph nodes. Scoring of gross and histopathological lesions was undertaken blinded without knowledge of the vaccine groups.

### IFN-γ assay

Heparinised blood samples were collected from the cattle at 6 monthly intervals using the standard New Zealand protocol for IFN-γ tesing and at more regular intervals to analyse the kinetics of IFN-γ responses. For the 6 monthly testing from 6 to 30 months following BCG vaccination, incubation of aliquots of blood and IFN-γ analyses were undertaken in the National Laboratory for bovine TB IFN-γ testing at AgResearch Wallaceville, while the IFN-γ kinetics studies were undertaken in the AgResearch Hopkirk laboratory. Blood samples (1.5 ml) were dispersed into wells of a 24-well plate and stimulated with preservative-free bovine purified protein derivative (PPD) prepared from *M. bovis* or PPD prepared from *M. avium* (22 µg/ml final concentration; Prionics, Schlieren-Zurich, Switzerland) or ESAT-6/CFP10 fusion protein (4 µg/ml final concentration; Statens Serum Institute) or PBS (Nil). For the 6 monthly testing, blood samples were set up for incubation after 24 to 30 hours following blood collection with blood samples collected at the time of the tuberculin intradermal inoculation. After incubation at 37°C for 18 to 24 hours, the plasma supernatants were harvested and the IFN-γ levels measured using an ELISA kit (Prionics). Positive responses were defined using the New Zealand standard, with a positive PPD IFN-γ test as bovine PPD optical density (OD) minus avian PPD OD ≥0.100 OD 450 nm and a positive in the ESAT-6/CFP10 IFN-γ test as antigen OD minus Nil (PBS) OD ≥0.040 OD 450 nm [23]. For studying the kinetics of IFN-γ release, blood samples were set up within 6 hours of blood collection, incubated at 37°C for 20 to 24 hours and plasma supernatants tested for IFN-γ in an ELISA using the Mabtech IFN-γ reagents (Stockholm, Sweden). Results were expressed as pg/ml of IFN-γ using a standard curve and results were presented as bovine PPD (pg/ml) minus Nil (PBS) (pg/ml) or ESAT-6/CFP10 (pg/ml) minus Nil (PBS) (pg/ml).

### Tuberculin intradermal test

The CFT was undertaken by an AsureQuality technician involved in the New Zealand bovine TB testing programme and was conducted at 6, 12, 18, 24 and 30 months after the initial BCG vaccination. For this test, cattle were inoculated intradermally in the caudal fold of the tail with a 0.1****ml volume containing 5,000 IU of bovine PPD (AsureQuality, Upper Hutt, New Zealand) and the caudal fold was palpated at 72 hours to detect any evidence of swelling in comparison with opposite caudal fold. If there was any swelling, callipers were used to quantify the difference between the thickness of the injected and opposite caudal folds. At 11 weeks following the *M. bovis* challenge, a comparative cervical intradermal test was conducted on the animals from the non-vaccinated, BCG once and BCG/BCG groups to compare responses induced by avian and bovine PPDs. For the comparative test, 0.1****ml volumes containing either 2,500 international units (IU) of avian PPD or 3,000 IU of bovine PPD (Prionics) were inoculated intradermally at separate sites on the right side of the neck. The skin-fold thickness was measured with callipers prior to and 72 hours after inoculation of the PPDs.

### Antibody ELISA

Blood samples were collected at regular intervals during the study to measure serological responses. Sera were stored at **−**20°C until they were tested. The *M. bovis* AN5 culture filtrate protein and ESAT-6 peptides (aa 1–16, 9–24, 17–32, 57–72, 80–95) and Ag85A peptides (aa 70–78, 99–118, 145–152; Auspep, Melbourne, Australia) were diluted to 3** µ**g/ml in carbonate buffer (pH 9.6); 100** µ**l per well was added to 96-well ELISA plates (Maxisorp; Nunc, Roskilde, Denmark) and plates incubated overnight at 4°C. The antibody ELISA was carried out as described previously [17]. The results were expressed as “absorbance indexes”, calculated by expressing the values found for the test sera as a fraction of the binding of a strong positive reference serum multiplied by 100. A strong positive reference serum was included in each plate and results for each plate were expressed as a percentage of this control to eliminate any plate to plate variations. The strong positive reference serum was composed of a pool of sera collected from *M. bovis*-experimentally-infected cattle from a previous trial where these animals had strong antibody responses to *M. bovis* antigens.

### Statistical analyses

The statistical analyses for antibody and IFN-γ responses were undertaken using R 3.0.0 software [24]. A mixed effects model was applied to natural log-transformed antibody and non-transformed IFN-γ responses; time, group and their interaction were fixed effects, and animal was a random effect. Fisher’s Exact test was used for comparing the proportion of animals with lung or lymph node lesions. For the remaining data, statistical analyses were undertaken using Minitab 16. Intradermal test data were analysed using the t-test. Lesions scores were compared using pair-wise Mann-Whitney U-test. The mean number of lesioned lymph nodes/animal or nodes culture positive for *M. bovis*/animal were compared using ANOVA with Tukey’s multiple comparisons. Statistical significance was denoted when *p*<0.05.

## Results

### Effect of BCG vaccination on standard diagnostic tests for bovine TB

Vaccination with BCG resulted in higher proportions of animals reacting positively in the tuberculin intradermal CFT and PPD IFN-γ tests, but not in the DIVA test, ESAT-6/CFP10 IFN-γ test, at 6 months after vaccination compared to those for the non-vaccinated animals, although the differences were not significant (Table 1). Whereas, at 12, 18 and 24 months after vaccination, the proportion of BCG-vaccinated animals reacting positively in the tuberculin intradermal CFT and PPD IFN-γ tests were the same or slightly lower than those for the non-vaccinated animals, again there were no significant differences between the two groups. The positive intradermal CFT responses in the period from 6 to 24 months were all small; median of 2.25 mm (range 1.5, 4) increase in fold thickness for the responding non-vaccinated reactor animals and median 2.0 mm (range 1, 4) for the responding BCG-vaccinated animals. Surprisingly, positive responses were observed in the ESAT-6/CFP10 IFN-γ test on 10 occasions from animals in the non-vaccinated and BCG-vaccinated groups in the first 24 months of the study (total of nine different animals; Table 1). In nine of the 10 cases, these positive reactions were associated with strong avian PPD-specific responses (median avian PPD response for the 10 cases was 0.725 OD 450 nm, range 0.043, 2.287). In all cases, the avian PPD response was greater than that for bovine PPD. At 30 months after the initial BCG vaccination which was 6 months after revaccination, five animals reacted positively in the tuberculin intradermal CFT (three from the BCG/BCG group and one each in the BCG/Biobeads and BCG/CFP groups). Two animals reacted positively in the PPD IFN-γ test (one each in the BCG/BCG and BCG/CFP groups and three in the ESAT-6/CFP10 IFN-γ test (one each in the BCG/BCG, BCG/Biobeads and BCG/CFP groups).

**Table 1:** Immunological responses in routine tuberculosis diagnostic tests following vaccination with BCG.

	Proportion of animals positive (%)					
Time after BCGvaccination(months)	Caudal foldtest^a^ ≥1 mm increase		PPD IFN-γ test^b^B-A ≥0.100 OD		ESAT-6/CFP10 IFN-γ test^c^Antigen-Nil ≥0.040 OD	
	NV	BCG	NV	BCG	NV	BCG
6	2/17	15/62	0/17	6/62	1/17	0/62
	(12%)	(24%)	(0%)	(10%)	(6%)	(0%)
12	3/17	6/62	0/17	0/62	0/17	1/62
	(18%)	(10%)	(0%)	(0%)	(0%)	(2%)
18	1/17	0/62	0/17	0/62	1/17	4/62
	(6%)	(0%)	(0%)	(0%)	(6%)	(6%)
24	0/17	0/62	0/17	0/62	0/17	3/62
	(0%)	(0%)	(0%)	(0%)	(0%)	(5%)

a Caudal fold skin test positive defined as ≥1 mm increase in skin thickness.

b PPD IFN-γ test positive defined as Bovine PPD OD – Avian PPD OD ≥0.100 OD.

c ESAT-6/CFP10 IFN-**γ** test positive defined as ESAT-6/CFP10 (Antigen) OD -Nil (PBS) OD ≥0.040 OD NV, non-vaccinated BCG, BCG-vaccinated.

### Immune responses following vaccination, revaccination and challenge

**Figure 1:**
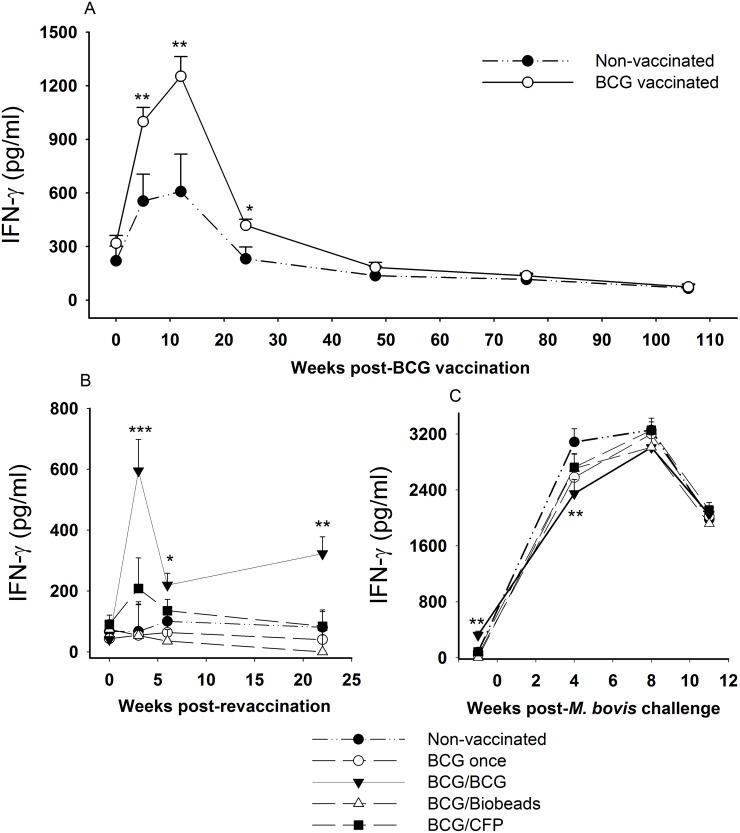
Bovine PPD-specific IFN-γ responses of cattle after vaccination, revaccination and *Mycobacterium bovis* challenge. Mean responses for groups of cattle vaccinated with BCG at 2 to 4 weeks of age (○, n = 62) and compared with non-vaccinated animals (•, n = 17) up to the time of revaccination at 106 weeks (A); mean responses for groups of cattle following revaccination (B); mean responses for groups of cattle following endotracheal challenge with *M. bovis* (C). Non-vaccinated cattle (•, n = 17); BCG-vaccinated, but not revaccinated (○, BCG once group, n = 16); BCG-vaccinated and revaccinated 2 years later with BCG (▾, BCG/BCG group, n = 15); BCG-vaccinated and revaccinated 2 years later with TB biobeads displaying mycobacterial proteins, ESAT-6 and Ag85A (Δ, BCG/Biobeads group, n = 15), BCG-vaccinated and revaccinated 2 years later with *M. bovis* culture filtrate protein vaccine (▪, BCG/CFP group, n = 16). Results expressed as IFN-γ bovine PPD (pg/ml) minus Nil (PBS) pg/ml. Error bar represented SEM. Significant difference from the non-vaccinated group was denoted by ***p<0.001, **p<0.01, *p<0.05.

**Figure 2:**
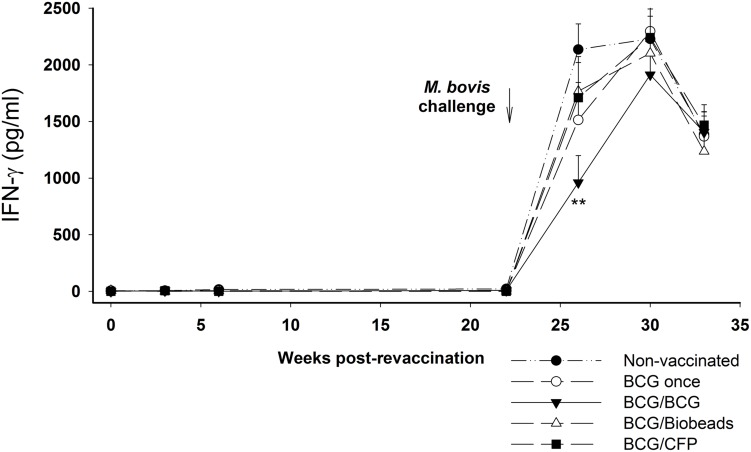
ESAT-6/CFP10-specific IFN-γ responses of cattle after revaccination and *Mycobacterium bovis* challenge. Mean IFN-γ responses for groups of cattle following revaccination and subsequent challenge with *M. bovis* at 23 weeks after revaccination. Non-vaccinated cattle (•, n = 17); BCG-vaccinated, but not revaccinated (○, BCG once group, n = 16); BCG-vaccinated and revaccinated 2 years later with BCG (▾, BCG/BCG group, n = 15); BCG-vaccinated and revaccinated 2 years later with TB biobeads displaying mycobacterial proteins, ESAT-6 and Ag85A (Δ, BCG/Biobeads group, n = 15), BCG-vaccinated and revaccinated 2 years later with *M. bovis* culture filtrate protein vaccine (▪, BCG/CFP group, n = 16). Results expressed as IFN-γ ESAT-6/CFP10 (pg/ml) minus Nil (PBS) pg/ml. Arrow indicated challenge with *M. bovis* at 23 weeks after revaccination and error bar represented SEM. Significant difference from the non-vaccinated group was denoted by **p<0.01.

The kinetics of bovine PPD-specific IFN-γ responses for the different groups are shown in Figure 1. The calves vaccinated with BCG at 2–4 weeks of age produced significantly stronger bovine PPD-specific IFN-γ responses than those for non-vaccinated animals at 5 and 12 weeks (*p*<0.01) and at 24 weeks after vaccination (*p*<0.05; Figure 1A). The relatively high mean bovine PPD response in the non-vaccinated group at 5 and 12 weeks was associated with a strong avian PPD-specific IFN-γ response (data not shown). Following revaccination with BCG at 2 years (BCG/BCG group), the bovine PPD-specific IFN-γ responses were significantly greater than those observed for the non-vaccinated animals at 3 weeks (*p*<0.001), 6 weeks (*p*<0.05) and 22 weeks after revaccination (*p*<0.01; Figure 1B). However, the increase in the mean IFN-γ response following BCG revaccination was smaller than that observed following the initial BCG vaccination. Revaccination of calves with the CFP vaccine (BCG/CFP group) resulted in a small, but non-significant increase in the bovine PPD-specific IFN-γ response compared to that for the non-vaccinated animals (Figure 1B). Revaccination with the TB Biobead vaccine (BCG/Biobeads group) did not result in an increase in the bovine PPD-specific (Figure 1B) or the ESAT-6/CFP10-specific IFN-γ response (Figure 2). Following challenge with *M. bovis*, all groups showed a marked increase in the bovine-PPD-specific (Figure 1C) and ESAT-6/CFP10-specific IFN-γ responses (Figure 2). The only significant differences between the groups after challenge were lower bovine PPD-specific and ESAT-6/CFP10-specific IFN-γ responses in the BCG/BCG group at 4 weeks post challenge (*p*<0.01).

**Figure 3:**
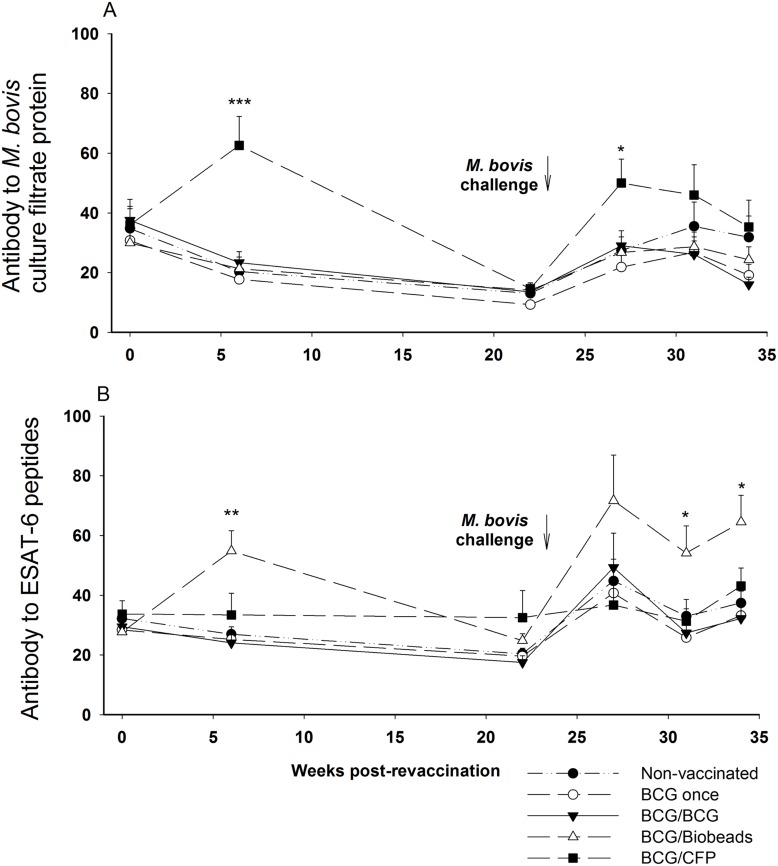
Antibody responses after revaccination and *Mycobacterium bovis* challenge. Antibody responses to *M. bovis* culture filtrate protein (A) and ESAT-6 peptides (B) were measured after revaccination and *M. bovis* challenge for the different vaccine groups. Non-vaccinated (•, n = 17); BCG-vaccinated, not revaccinated (○, BCG once group, n = 16); BCG-vaccinated and revaccinated 2 years later with BCG (▾, BCG/BCG group, n = 15); BCG-vaccinated and revaccinated 2 years later with TB biobeads displaying mycobacterial proteins, ESAT-6 and Ag85A (Δ, BCG/Biobeads group, n = 15), BCG-vaccinated and revaccinated 2 years later with *M. bovis* culture filtrate protein vaccine (▪, BCG/CFP group, n = 16). All animals were challenged with *M. bovis* at 23 weeks after revaccination (129 weeks after the initial BCG vaccination). Results expressed as a percentage of a strong positive control. Significantly different from the non-vaccinated group was denoted by ***p<0.001, **p<0.01, *p<0.05, with analysis undertaken on natural log-transformed data.

Serum antibody responses to *M. bovis* culture filtrate protein and ESAT-6 and Ag85A peptides were measured for all groups following revaccination and challenge. Revaccination with the CFP vaccine (BCG/CFP group) induced a significant increase in antibody responses to *M. bovis* culture filtrate protein compared to that for the non-vaccinated animals at 6 weeks following the first CFP vaccination (*p*<0.001) and at 4 weeks after the *M. bovis* challenge (27 weeks after revaccination, *p*<0.05; Figure 3A). Furthermore, revaccination with the Biobead vaccine (BCG/Biobeads group) induced significant increases in antibody to ESAT-6 peptides compared to that for the non-vaccinated group at 6 weeks after the first Biobead vaccination (*p*<0.01), at 8 and 11 weeks following *M. bovis* challenge (*p*<0.05; Figure 3B). Compared to the non-vaccinated group, none of the vaccinated groups showed a significant antibody response to Ag85A (data not shown). At the final bleed prior to slaughter of the animals, 11 weeks post-challenge (34 weeks post-revaccination), the antibody response to *M. bovis* CFP for the BCG/BCG group was lower than that of the non-vaccinated group, but not significantly (*p* = 0.058).

The tuberculin comparative cervical intradermal test was conducted on the non-vaccinated, BCG once and BCG/BCG groups at 11 weeks after *M. bovis* challenge. All cattle responded strongly to bovine PPD, with responses ranging from 5 to 30 mm increase in skin thickness and responses to bovine PPD were all greater than those for avian PPD. Only one animal had a bovine PPD minus avian PPD response of ≤4 mm. This animal was from the BCG/BCG group and this was the only animal from these groups from which no *M. bovis* was isolated. The mean (±SEM) increases in skin thickness for the non-vaccinated, BCG once and BCG/BCG groups for bovine PPD were 17.6 (±1.6), 17.5 (±1.0) and 16.3 (±1.4) mm and for avian PPD, 4.1 (±0.6), 4.7 (±0.6) and 3.6 (±0.7) mm, respectively. No significant differences were observed between the groups for responses to bovine or avian PPD.

### Protection against challenge with *M. bovis*

The endotracheal challenge with *M. bovis* produced gross tuberculous lesions in the lungs and pulmonary lymph nodes in the majority of the cattle and no detectable lesions were observed in lymph nodes and organs outside the pulmonary cavity. The lesions were typical of those for bovine TB in cattle with multiple small (1–3 mm in diameter) caseous or calcified lesions in the lung and variable sized calcified lesions in the pulmonary lymph nodes (1–20 mm in diameter). All of the gross lesions in the lungs and pulmonary lymph nodes were confirmed as tuberculous lesions by culture of *M. bovis* from the tissues.

**Figure 4:**
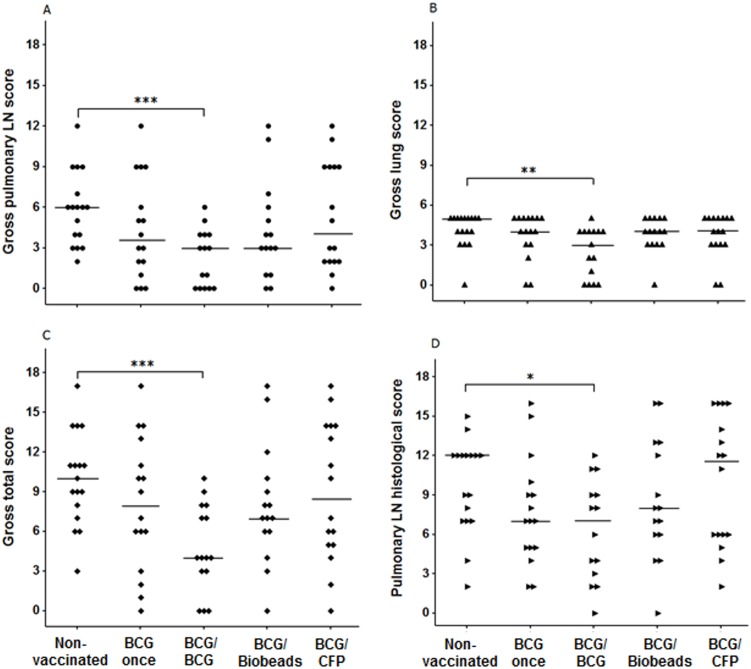
Gross and histopathological lesion scores of cattle following *Mycobacterium bovis* challenge. Gross pulmonary lymph node (LN) lesion score (A), lung lesion score (B), total lesion score (pulmonary lymph node and lung scores combined) (C) and pulmonary lymph node histopathological lesion score (D). Scores were determined as described in the Material and Methods and scores for individual animals were plotted. Non-vaccinated; BCG once, BCG-vaccinated, not revaccinated; BCG/BCG, BCG-vaccinated and revaccinated 2 years later with BCG; BCG/Biobeads, BCG-vaccinated and revaccinated 2 years later with TB biobeads displaying mycobacterial proteins, ESAT-6 and Ag85A; BCG/CFP, BCG-vaccinated and revaccinated 2 years later with *M. bovis* culture filtrate protein vaccine. Median indicated by horizontal line. Significant difference from the non-vaccinated group was denoted by ***p<0.001, **p<0.01 and *p<0.05.

**Figure 5:**
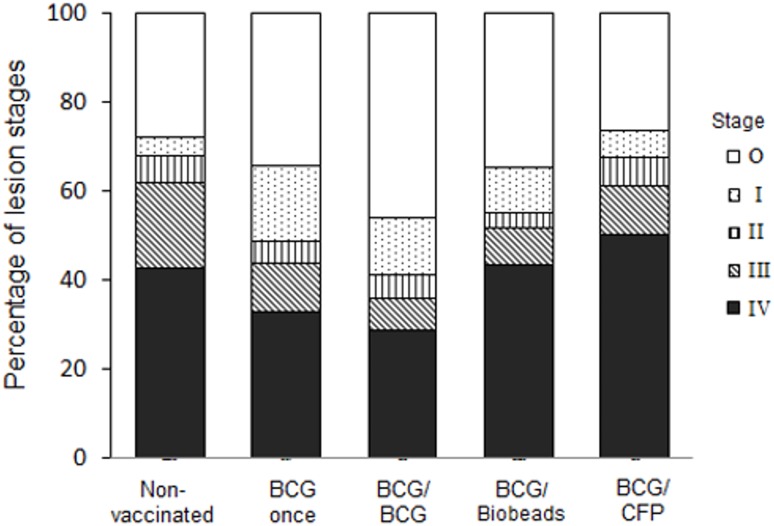
Histological evaluation of pulmonary lymph nodes following challenge with *Mycobacterium bovis*. Sections were stained with hematoxylin and eosin and the most severe stage of granuloma in each section was determined as described in the Material and Methods. The stage of granuloma was indicated by Stage 0 (no granuloma, white bars), Stage I (dotted bars), Stage II (vertically hatched bars), Stage III (diagonally hatched bars) and Stage IV (black bars). Data were shown as proportions for each stage. Non-vaccinated; BCG once, BCG-vaccinated, not revaccinated; BCG/BCG, BCG-vaccinated and revaccinated 2 years later with BCG; BCG/Biobeads, BCG-vaccinated and revaccinated 2 years later with TB biobeads displaying mycobacterial proteins, ESAT-6 and Ag85A; BCG/CFP, BCG-vaccinated and revaccinated 2 years later with *M. bovis* culture filtrate protein vaccine. The proportion of Stage III and IV granulomas combined for the BCG once and BCG/BCG groups were significantly less than that for the non-vaccinated group (*p*<0.05).

Protection against challenge with *M. bovis* was assessed from comparisons between groups of the proportion of animals with gross pulmonary lymph node or lung lesions, mean number of lesioned or *M. bovis*-infected pulmonary lymph nodes per animal (Table 2) and pulmonary and lung lesion scores (Figure 4). The BCG/BCG group had significant reductions in the proportion of animals with gross pulmonary lymph node lesions (*p* = 0.015) and mean number of lesioned pulmonary lymph nodes per animal (*p* = 0.012) compared to those for the non-vaccinated group (Table 2). In addition, the BCG/BCG group had a significantly lower pulmonary lymph node lesion score (*p*<0.001), lung lesion score (*p* = 0.007), total lesion score (*p*<0.001) and histopathological lesion score (*p* = 0.018) compared to those for the non-vaccinated group (Figure 4). No significant reductions in these parameters were observed in the three other vaccinated groups in comparison with the non-vaccinated group. The grading of the histopathological lesions was based on the most severe lesion observed in the section and many sections contained all four stages of granulomas. Stage III and IV granulomas contained the largest amount of necrotic material and both the BCG once and BCG/BCG groups had a significantly lower proportion of these two types of granulomas combined compared to that for the non-vaccinated group (*p*<0.05; Figure 5). *M. bovis* was cultured from a lung or pulmonary lymph node sample from all but two animals; one from each of the BCG/BCG and BCG/Biobeads groups.

**Table 2:** Summary of pathological and bacteriological findings following *Mycobacterium bovis* challenge.

Vaccine Group	Proportions withlesions in		No. of lesioned LNs/animal(mean ± SEM)	No. of *M. bovis* culture-positiveLNs/animal (mean ± SEM)
	LN	Lung		
Non-vaccinated	17/17	16/17	2.76±0.22	3.00±0.24
BCG once	13/16	14/16	1.75±0.33	2.94±0.21
BCG/BCG	10/15*	11/15	1.33±0.30*	2.33±0.25
BCG/Biobeads	13/15	14/15	1.93±0.33	2.67±0.33
BCG/CFP	15/16	14/16	2.38±0.33	3.00±0.27

LN lymph node.

Non-vaccinated; BCG once, BCG-vaccinated, not revaccinated; BCG/BCG, BCG-vaccinated and revaccinated 2 years later with BCG; BCG/Biobeads, BCG-vaccinated and revaccinated 2 years later with TB biobeads displaying mycobacterial proteins, ESAT-6 and Ag85A; BCG/CFP, BCG-vaccinated and revaccinated 2 years later with *M. bovis* culture filtrate protein vaccine. Cattle were challenged with *M. bovis* 129 weeks after initial BCG vaccination and 23 weeks after revaccination. All animals were slaughtered and examined for TB lesions at 13 weeks after challenge. *Significantly different from the non-vaccinated group, *p*<0.05.

## Discussion

The incomplete protection observed following BCG vaccination of cattle has limited its application for control of bovine TB and similarly, the variability of protection following BCG vaccination in humans has been an impediment for the control of TB in humans. Knowledge of the duration of protection against TB induced by BCG vaccination in cattle and the effect of revaccination has the potential to improve strategies for the use of this vaccine in cattle as well as to provide insights for the use of BCG in humans. The results from the current study have shown that protection against bovine TB had waned when cattle were challenged 2½ years following vaccination which concurred with an earlier study when protection against TB was reduced at 2 years after vaccination compared to protection after 1 year [15]. In both studies, the median total lesion score for animals challenged at 2 or more years after BCG vaccination was slightly lower than those observed in the respective control groups, although the differences did not reach significance, even with the larger group sizes utilised in the current study (n = 15–17 animals/group). The failure to observe statistically significant differences in the current study, with the exception of the lower proportion of more advanced histopathological lesions (Stage III and IV granulomas), was in part due to the large variation in responses within the single BCG-vaccinated group. Since Stage III and IV granulomas contain moderate to large numbers of acid-fast bacilli [2], the reduction in these types of granulomas in the group receiving BCG vaccine 2½ years prior to challenge could result in the reduction of spread of infection between cattle.

The experimental endotracheal challenge with *M. bovis* is more severe than that observed following natural exposure as it is aimed to produce highly reproducible pathology in the majority of the non-vaccinated animals, although mimicking the pathology observed in naturally-infected animals. A natural exposure to *M. bovis* would be expected to be a less severe challenge and hence, the duration of protection induced by BCG vaccine is likely to be greater in the field. There was an indication that protection against TB extended out to 23 months in a recent field BCG vaccination study in Ethiopia [25].

Revaccination of cattle at 2 years after the initial vaccination when both the tuberculin intradermal CFT and PPD IFN-γ test responses had waned was shown to significantly reduce the gross pulmonary lymph node, lung and total lesion scores as well as the histopathological lesion score. In contrast, a previous BCG revaccination study in young calves, demonstrated that protection was significantly reduced in those vaccinated as neonates and again at 6 weeks of age in comparison to those only vaccinated as neonates [26]. Interestingly, in that study, the revaccinated calves with greatest pathology had the strongest peripheral blood antigen-specific IFN-γ and IL-2 responses after vaccination. This indicated that an inappropriate immune response had been induced in these calves when revaccinated at a time when they had a strong pre-existing immune response to BCG. To fully investigate the effect of revaccinating cattle with BCG, there are a number of different combinations which could be tested. Additional groups could include a single vaccination at 2 years of age to determine whether similar protection was induced compared to those vaccinated with BCG within 1 month of age and again at 2 years, or for those revaccinated with BCG after 1 year. The rationale for the current study was to determine whether protection persisted for up to 2½ years post-vaccination and could immunity be boosted when immunity had waned.

Revaccination of other animal species with BCG has produced conflicting results. Subcutaneous BCG revaccination of 5–6 month old deer calves at an interval of 4–8 weeks enhanced protection compared to a single vaccination, whereas increasing this period to 43 weeks largely ablated protection [27]. In brushtail possums, application of BCG by intranasal spray and conjunctival instillation on 12 occasions at weekly intervals induced a greater level of protection compared to those receiving a single vaccination or revaccination after a period of 6 weeks [28]. In humans, revaccination with BCG may be more effective in temperate climates where there was a lower exposure to environmental mycobacteria. Revaccination gave an average 19% protection in coastal Salvador over 9 years of follow-up, while there was no evidence of protection in equatorial Manaus [29]. Revaccination of children and adults in Karonga District of Malawi induced no protection against TB [30] and no protection against TB was observed following revaccination of 6–9 year old children in Hong Kong [31]. In contrast, there were fewer infections (measured as positive responses on a Quanti-FERON TB gold test) among prison inmates in Taiwan who had greater than or equal to two BCG scars, when compared with those who had one scar or no scar [32]. Studies in cattle support the hypothesis that revaccination should be effective when immunity has waned and concur with the idea that revaccination of humans may be effective in locations where human populations do not respond in the tuberculin intradermal test [9].

Although previous studies have shown that co-administration of BCG and CFP vaccines to cattle enhanced protective immunity compared to administration of BCG alone [17], [18], revaccination with TB protein vaccines, CFP or Biobead vaccine, was not effective. This failure is probably due to the weak or non-existent antigen-specific IFN-γ responses induced following revaccination with the TB protein vaccines, while the vaccines were shown to be immunogenic from the induction of antibody responses. This was further reinforced by the anamnestic boost in antibody responses to *M. bovis* culture filtrate protein or ESAT-6 peptides following *M. bovis* challenge observed in the two respective groups revaccinated with the TB protein vaccines. High priorities for development of effective TB protein vaccines for cattle should be selection of appropriate adjuvants and immunomodulators to induce strong cellular immune responses as well as optimising the antigenic dose. In a subsequent study testing the TB Biobead vaccine in cattle, antigen-specific IFN-γ responses were enhanced by increasing the antigenic dose (N. Parlane, unpublished observations).

Correlates of protection observed in the study were the significantly lower IFN-γ responses to both bovine PPD and ESAT-6/CFP10 observed post-challenge in the BCG/BCG group compared to those in the non-vaccinated group. Others have reported robust or increasing ESAT-6/CFP10-specific IFN-γ responses in cattle after challenge are generally a negative prognostic indicator of vaccine efficacy and positively correlate with TB-associated pathology [33], [34].

At 6 months following vaccination with BCG, there were higher proportions of animals responding in the routine TB diagnostic tests (CFT and PPD IFN-γ test) compared to the non-vaccinated group, although the differences were not significant. However, the proportions of responding BCG-vaccinated animals at 12, 18 and 24 months after vaccination were the same or less than those in the non-vaccinated animals and the BCG-vaccinated animals did not respond in the DIVA, ESAT-6/CFP10 IFN-γ test at 6 months after vaccination. A small proportion of the cattle revaccinated with BCG (three of 15 animals) produced positive tuberculin intradermal CFT when tested 22 weeks after revaccination. In a UK study, the proportion of BCG-vaccinated animals responding in the SICCT at 6 months after vaccination was markedly higher than that observed in the CFT from the current study [5]. This effect is not likely to be due to the 5-fold lower dose of BCG Danish used for vaccination as a comparison of the higher dose and a 10-fold lower dose in cattle have induced similar SICCT responses and protection against challenge with *M. bovis* [35]. The CFT should have a higher sensitivity than the SICCT as the CFT measures any detectable response to bovine PPD, while the SICCT measures the difference between bovine and avian PPD responses (B– A) with positive cut-offs of B – A of >4 mm standard and B – A >2 mm severe interpretation. In the current study there was a high exposure to environmental mycobacteria with high IFN-γ responses to avian PPD (data not shown) also seen in the CFT responses observed in the non-vaccinated animals. Studies in humans have suggested that the more rapid decline in tuberculin responsiveness following BCG vaccination in individuals in tropical rather than temperature climates may be a consequence of their higher exposure to other infections including environmental mycobacteria [36]. This is most likely the reason for the relatively small number of BCG-vaccinated cattle responding in the CFT at 6 months and similarly, only a small number of BCG-vaccinated cattle responded in SICCT in a Malawi study [37].

Use of the DIVA tests such as the ESAT-6/CFP10 IFN-γ test will be important to allow BCG vaccine to be used in cattle and still retain the ability to differentiate BCG-vaccinated from those infected with *M. bovis*. However, the current study has emphasised the benefit of using the combination of the PPD and ESAT-6/CFP10 IFN-γ tests for bovine TB diagnosis as some positive ESAT-6/CFP10 IFN-γ responses in non-infected animals can arise from exposure to some environmental mycobacteria [38] or to activation of NK cells in young animals [39].

In summary, calves vaccinated with BCG at 2 to 4 weeks of age did not show a significant level of protection against an experimental challenge with *M. bovis* 2½ years later, other than a reduction in the more severe Stage III and IV granulomas. In contrast, a similar group of animals which were revaccinated with BCG at 2 years of age displayed a significant reduction in TB lesion scores in comparison to non-vaccinated animals, while revaccination with either of two TB protein vaccines failed to induce protection. The study has provided encouragement that protection against TB in cattle can be enhanced by revaccination with BCG when immunity had waned and further studies are now required to optimise the timing of revaccination and to evaluate BCG revaccination in a field situation.
